# How nature nurtures: prenatal exposure to green space buffers the effects of maternal stress on neonatal *BDNF* methylation

**DOI:** 10.1038/s41380-025-03379-1

**Published:** 2025-12-13

**Authors:** Sarah Nazzari, Grazia Zulian, Serena Grumi, Enrico Pisoni, Roberto Bergamaschi, Roberto Giorda, Renato Borgatti, Livio Provenzi

**Affiliations:** 1 Department of Brain and Behavioral Sciences, University of Pavia, Pavia, Italy; 2 Physical Geography and Landscape Ecology Section, Institute of Earth System Sciences, Leibniz University Hannover, Hannover, Germany; 3 Developmental Psychobiology Lab, IRCCS Mondino Foundation, Pavia, Italy; 4 European Commission, Joint Research Centre (JRC), Ispra, Italy; 5 Multiple Sclerosis Center, IRCCS Mondino Foundation, Pavia, Italy; 6 Molecular Biology Lab, Scientific Institute IRCCS E. Medea, Bosisio Parini, Italy

**Keywords:** Predictive markers, Molecular biology

## Abstract

Pregnancy constitutes a critical window of vulnerability during which maternal and environmental exposures may shape fetal development through epigenetic mechanisms. While prenatal maternal anxiety and exposure to green spaces have been independently associated with child neurodevelopment, their potential interactive effects on neonatal epigenetic profiles remain largely unexplored. This study examined the independent and interactive effects of maternal trait anxiety and residential green space exposure during pregnancy on neonatal DNA methylation (DNAm) of the brain-derived neurotrophic factor (*BDNF*) gene. A sample of 110 mother-infant dyads was enrolled at delivery. Maternal trait anxiety was assessed using the Stait-Trait Anxiety Inventory (STAI-Y) and infants’ *BDNF* DNAm at birth was assessed in 11 CpG sites in buccal cells. Prenatal residential addresses were geocoded and green space availability within 300, 500, and 1000 m buffers was calculated using the CLCplus Backbone 2021 land cover dataset. Hierarchical linear regression models were adjusted for infant sex and prenatal exposure to PM2.5. Results indicated that higher maternal trait anxiety was associated with increased BDNF DNAm at four CpG sites only among infants with lower exposure to green space within a 300 m buffer. This association was not significant at higher levels of greenness, suggesting a neuroprotective effect of natural environments during gestation. Findings provide novel evidence that urban green space may buffer the biological impact of maternal anxiety on neonatal *BDNF* methylation. This highlights the importance of integrating psychological and environmental-level exposures to elucidate early-life determinants of neurodevelopment.

## Introduction

The critical role of early-life environments in influencing neurodevelopmental trajectories and long-term health outcomes is now widely acknowledged [1]. Within this developmental continuum, pregnancy stands as a period of exceptional susceptibility to direct environmental exposures [2]. Adverse exposures during gestation, such as stress, infections or toxins, can induce lasting alterations in fetal neurobiological systems, with potential consequences for cognitive, emotional, and behavioral outcomes across the lifespan [3–5]. Amassing evidence highlights the significant impact of antenatal maternal emotional distress, encompassing symptoms of stress, depression and anxiety, on the developing fetus, showing associations with a range of child developmental outcomes [6]. The last decade has also seen a growing appreciation for the influence of the physical environment, including air pollution, noise and green spaces, in shaping individuals’ neurodevelopmental trajectories [7–9]. This expanding literature highlights the multifaceted nature of prenatal influences and compels a comprehensive research approach that integrates these diverse environmental exposures to investigate their cumulative and interactive role in developmental outcomes.

Epigenetic modifications, particularly DNA methylation (DNAm), are increasingly recognized as critical mechanisms mediating the effects of intrauterine conditions on neurodevelopmental outcomes [10, 11]. DNAm refers to the addition of a methyl group to the cytosine DNA base in a cytosine nucleotide–phosphate–guanine nucleotide (CpG) sequence, often resulting in altered gene expression [12]. The current study extends existing literature by investigating the joint impact of prenatal maternal anxiety and exposure to urban green space on DNAm of the Brain-Derived Neurotrophic Factor (*BDNF*) gene in neonates. BDNF is a key neurotrophin in the Central Nervous System (CNS), regulating cellular processes essential for brain development and function, including neurogenesis and synaptic plasticity [13]. Furthermore, mounting evidence suggests a role for BDNF deficiency, including DNAm of the *BDNF* gene, in the pathophysiology of several psychiatric disorders [14–18]. Animal studies consistently support the role of *BDNF* epigenetic modifications in mediating the effects of prenatal adversity on neurobehavioral trajectories [19]. In contrast, human research is limited, and findings are mixed. For example, Kertes et al. [20] observed increased *BDNF* DNAm in cord blood and placental samples of 24 mother-infant dyads exposed to war-related traumas. Likewise, Nazzari et al., [21] found that higher maternal trait anxiety predicted greater *BDNF* DNAm but only in males newborns. In contrast, Braithwaite et al. [22] reported a negative association between antenatal depressive symptoms and *BDNF* DNAm in 2-month-old infants, while Devlin et al. [23] found no associations between maternal depression or antidepressant use during pregnancy and *BDNF* DNAm in cord blood. These inconsistencies highlight the need to clarify whether increased *BDNF* DNAm can serve as a biomarker of prenatal adversity in humans. Importantly, such discrepancies may reflect unmeasured co-occurring prenatal environmental factors interacting to shape epigenetic outcomes. Moreover, while studies have considered a broad spectrum of maternal psychological conditions, these differ substantially in their physiological signatures and potential effects on fetal development. In the current study, we focused on maternal trait anxiety, a relatively stable dispositional tendency to experience chronic worry and hyperarousal [24]. Trait anxiety has shown high temporal stability across pregnancy [25] and has been associated with specific biological stress-related alterations in pregnant women [26–28]. As such, it may constitute a consistent and biologically salient stress exposure for the fetus. Although less frequently investigated than depression, recent meta-analytical evidence suggests that antenatal anxiety is independently linked to several developmental outcomes including difficult infant temperament, behavioral problems, and poorer cognitive and language development [29].

Exposure to green space is consistently emerging as a protective factor for psychological well-being across the lifespan [30–32], including the prenatal period [33, 34]. Beyond mental health benefits, green space exposure has been associated with improved pregnancy and birth outcomes [35, 36]. Recently, a growing body of research has begun to examine links between green space exposure and DNAm [37, 38], with an emerging focus on the prenatal period as a critical window of epigenetic plasticity. A candidate gene study reported a positive association between residential green space within 1000 – 3000 m buffers and placental DNAm of the serotonin receptor *HTR2A* [39]. An epigenome-wide study showed that residential greenness within a 500 m buffer was associated with differential placental DNAm of the *SLC25A10* gene, involved in mitochondrial functions [40]. Alfano et al. [41] identified differentially methylated regions (DMRs) in cord blood associated with green space exposure, with stronger effects within the 100 m buffer compared to 1000 m. However, a recent meta-analysis found no robust associations between green space exposure and genome-wide DNAm levels over 400,000 CpG sites in cord or child blood [42]. These mixed findings highlight the need for further research into the epigenetic mechanisms through which green space may influence early development, with particular attention to possible interactions with co-occurring prenatal stressors.

Building on this literature, the present study investigates the independent and interactive effects of maternal trait anxiety and exposure to green space in close proximity to the home during pregnancy on neonatal *BDNF* DNAm. Prior research has examined maternal distress and green space separately, but their interactive influence on neonatal DNAm remains underexplored. This study addresses this gap by testing whether exposure to green space during pregnancy buffers the association between maternal anxiety and *BDNF* DNAm at birth. We predicted higher *BDNF* DNAm in infants exposed to greater maternal anxiety and lower residential greenness and hypothesized that greater green space availability would mitigate this association. We focused on *BDNF* given its relevance for neurodevelopmental and psychiatric outcomes [43]. Maternal trait anxiety was assessed after delivery and considered a proxy for chronic prenatal stress exposure as in previous studies [21, 44]. Green space was defined as any vegetated urban land, either public or private, including parks, tree-lined streets, recreational areas, and gardens. To capture individuals’ immediate environmental exposure, green spaces were quantified using 300, 500 and 1000 m circular buffers around each residence. These distances are informed by previous research showing that buffers within 500–999 m show the strongest associations with health outcomes [45].

## Methods and materials

### Participants and procedures

As part of the longitudinal, multi-center MOM-COPE Study [46, 47], women were enrolled between May 2020 and February 2021 during childbirth classes or shortly after delivery in ten neonatal units across Northern Italy. Inclusion criteria were: maternal age >18 years, term delivery (37 + 0 to 41 + 6 weeks), absence of prenatal/perinatal diseases, and a negative SARS-CoV-2 PCR test at delivery. A total of 297 mother-infant dyads provided complete data on maternal anxiety and infant DNA methylation. In a follow-up (Oct–Nov 2024), 110 women (37%) reported their residential address during pregnancy. The final sample size was consistent with an a priori power analysis conducted for detecting an interaction effect in a hierarchical regression model. The analysis indicated that 92 participants were required, assuming α = 0.05, power = 0.80, and a medium effect size (f = 0.15). Our final sample (N = 110) therefore exceeded the minimum required sample size, ensuring adequate power for the planned analyses. Participants and non-participants in the follow-up did not differ on sociodemographic, anxiety, or methylation variables (p ≥ 0.15). Sociodemographic (i.e., maternal age, education, occupation) and neonatal data (i.e., gestational age, birth weight, Apgar scores, breastfeeding, delivery mode, etc.) were obtained from medical records. Maternal trait anxiety was assessed within 48 h postpartum. Green space exposure was quantified geospatially using the prenatal address. Infant *BDNF* DNAm was measured from buccal cells collected shortly after birth. The study was approved by the Ethics Committees of the IRCCS Mondino Foundation and the participating hospitals and conducted in accordance with the 2018 Declaration of Helsinki. Written informed consent was obtained from all participants.

### Measures

*Maternal trait anxiety*. Maternal anxiety was assessed using the Italian adaptation [48] of the Trait subscale of the State-Trait Anxiety Inventory (STAI-T [49]). This 20-item self-report questionnaire evaluates the general tendency to experience anxiety (e.g., “I am a steady person” and “I lack self-confidence”), using a 4-point Likert scale from “almost never” to “almost always”. Total scores range from 20 (low anxiety) to 80 (high anxiety). The STAI-T shows strong test–retest reliability (stability coefficients between 0.73 and 0.86) [50] and high temporal stability during the perinatal period (r = 0.86 between weeks 28 and 38 and up to two years after childbirth) [25].

**Fig. 1:**
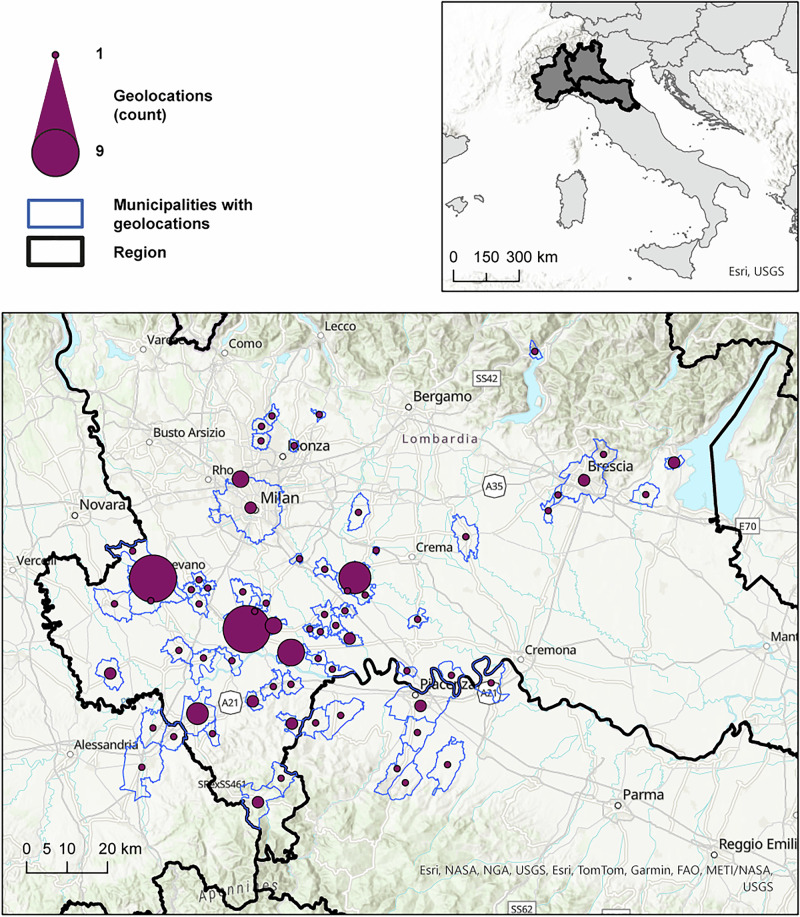
Graphical representation of the geographical distribution of geocoded residential addresses for participants across municipalities in the Lombardy region in Northern Italy. Each purple circle represents one or more geolocations, with circle size proportional to the number of participants residing in each municipality (range: 1–9). Municipal boundaries with at least one geolocation are outlined in blue, while the region’s boundary is highlighted in black.

*Antenatal exposure to green space*. Residential addresses were geocoded using the ArcGIS Pro Geocode Tool (Fig. 1). Green space availability within 300, 500 and 1000 m buffers was derived using the CLCplus Backbone 2021 land cover dataset. Green space exposure was operationalized as the percentage of land classified as green space, based on CLCplus categories. The following land types were included in main analyses: 1) *Tree cover* (needle-leaved and broadleaved trees), 2) *Low-growing vegetation* (shrubs, herbaceous plants, lichens and mosses), 3) *Total green space* (sum of all vegetation types), and 4) *Artificial land cover* (sealed surfaces). Spatial analyses were performed using ArcGIS Pro and GRASS GIS 8. All participants reported a single, stable address during pregnancy. See Supplementary Materials for details.

*Antenatal exposure to air pollution*. Based on residential addresses, fine particulate matter (PM_2.5_) ground-level concentrations were derived using data from the Copernicus Atmospheric Monitoring Service (CAMS) [https://atmosphere.copernicus.eu/]. Monthly average PM2.5 concentrations were spatially and temporally averaged and total exposure during pregnancy was calculated based on birth date [51]. This measure was included as a confounder in the analyses.

**Fig. 2:**
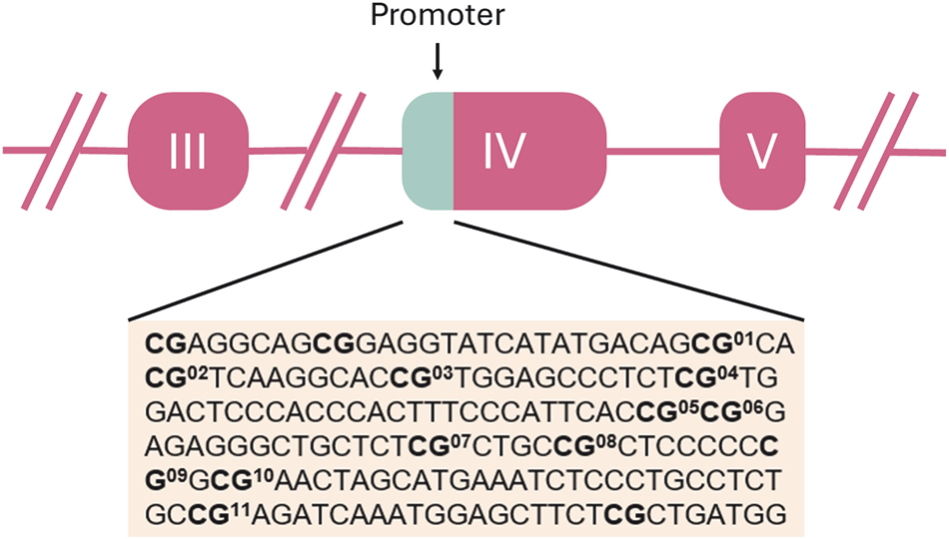
Schematic representation of the *BDNF* gene with the target sequences investigated (in light green). The pink boxes represent exons and the lines indicate introns. The position of the target sequence in the DNA and the exact base sequences is given below. In Bold CpG sites with the corresponding CpG unit number.

*Infant BDNF DNAm*. Between 6–24 h post-delivery, buccal cells were collected using the OC-175 OraCollect kit (DNA Genotek, Ottawa, Canada). The genomic DNA was extracted and assessed for quality using a Qubit fluorometer (Invitrogen, Thermo Fisher Scientific, Waltham, Massachusetts, USA). DNAm at 11 CpG sites in the *BDNF* promoter region (chr11: 27,723,096–27,723,219; Table 1 and Fig. 2) was assessed by PCR amplification of bisulfite-treated DNA followed by Next Generation Sequencing on a NEXTSeq500 (Illumina, San Diego, California, USA). This region was selected based on prior associations with antenatal adversity [20]. For details see Supplementary Materials.

**Table 1:** Positions of the selected *BDNF* CpG sites human genome assembly GRCh37 (hg19).

CpG site #	Position	% methylated Mean [Range]
1	Chr11: 27,723,218–27,723,219	0,64% [0,12–2,22]
2	Chr11: 27,723,214–27,723,215	0,42% [0,00–2,82]
3	Chr11: 27,723,203–27,723,204	0,49% [0,08–2,36]
4	Chr11: 27,723,190–27,723,191	0,34% [0,00–2,63]
5	Chr11: 27,723,161–27,723,162	0,54% [0,07–1,81]
6	Chr11: 27,723,159–27,723,160	0,41% [0,00–2,14]
7	Chr11: 27,723,143–27,723,144	0,39% [0,00–1,30]
8	Chr11: 27,723,137–27,723,138	0,47% [0,07–3,17]
9	Chr11: 27,723,128–27,723,129	0,45% [0,00–1,53]
10	Chr11: 27,723,125–27,723,126	0,51% [0,07–1,30]
11	Chr11: 27,723,095–27,723,096	0,48% [0,00–1,19]

### Data reduction

Distribution of *BDNF* DNAm levels at each CpG site was examined to exclude invariable sites. A threshold of SD < 0.05 or < 5% methylation was employed [52, 53]. All selected sites demonstrated individual-level variability (>20%) and were retained in the analysis. To reduce the number of CpG sites, a Principal Component Analysis (PCA) was performed [54, 55]. The PCA was carried out setting a varimax rotation, suppressing coefficients <0.30, and extracting principal components (PCs) based on eigenvalues > 1. A four-component solution provided the optimal fit (Supplementary Table 1). PC1 (composed of 4 CpG sites) and PC2 (composed of 3 CpG sites) accounted, respectively, for 22.4 and 20% of the variance in newborns *BDNF* DNAm and were used in further analyses.

### Plan of analysis

Variables were screened for outliers and skewness. Positively skewed distributions (i.e., methylation levels at each CpG site, indices of green space and pollution exposures) were natural log transformed. Samples >3 SD from the mean (N = 3) were removed. Covariates were selected via preliminary bivariate correlations and t-tests or ANOVA. Hierarchical linear regressions tested the independent and interactive effects of maternal anxiety and greenness on newborns’ DNAm (PC1 and PC2). Significant interactions were examined with simple slope analyses. Continuous variables were mean-centered, while infant sex was centered at males. Statistical analyses were performed using Jamovi 2.5.6 (The Jamovi Project, 2021).

## Results

### Preliminary analyses

Descriptive statistics are presented in Table 2, and bivariate correlations among the study variables are detailed in Supplementary Table 2. Of the 110 infants included in the study (N = 49 males), 5.6% were born in winter (December–February), 20.6% in spring (March–May), 59.8% in summer (June–August), and 14% in autumn (September–November).

**Table 2:** Sample characteristics.

	Min	Max	Mean	SD
Gestational age (weeks)	37	42	39.9	1.03
Birth weight (grams)	2480	4435	3330	380.0
Apgar at minute 1	5	10	9.12	0.79
Maternal age (years)	24	51	33.8	4.51
Maternal education (years)	8	22	15.6	3.20
Maternal trait anxiety	24	60	38.4	7.87

To assess the influence of sociodemographic (e.g., parental age, education) and perinatal health variables (e.g., birth weight, gestational age, Apgar scores) on infant *BDNF* DNAm levels, Pearson’s correlations were conducted. No significant associations emerged (all ps > 0.17), so these variables were excluded from further models. Independent samples t tests tested for sex differences in *BDNF* DNAm levels. A non-significant difference was observed for PC 1 (*t*(102) = 1.75, *p *= 0.08) with slightly higher DNAm in females. Given prior evidence of sex-dependent variations in newborns’ DNAm [21, 54], infant’s sex was included as a covariate. We also explored whether season of birth was associated with *BDNF* DNAm levels. No significant differences emerged across seasons (ANOVA for PC1: *F*(3, 105) = 0.18, *p* = 0.91; PC2: *F*(3, 105) = 0.10, *p* = 0.97), and this variable was not retained in further analyses. Total green space within all buffers was significantly and negatively associated with PM2.5 levels (rs range -0.22 to -0.38), thus models were adjusted for prenatal PM2.5. To further assess the potential confounding role of sociodemographic variables, we examined whether maternal age and maternal education were associated not only with *BDNF* methylation but also with the key predictors in our model (i.e. maternal anxiety and residential greenness). Pearson’s correlations revealed no statistically significant associations between maternal age or education and any of these variables (all *p*s > 0.09), suggesting limited risk of collinearity or confounding. To ensure that the reported effects were robust to sociodemographic adjustment, we reported results for the regression models including also maternal age and education as covariates in the Supplementary Analyses section of the Results.

Lastly, to better account for the contextual influence of the COVID-19 pandemic, we computed at which trimesters of pregnancy participants experienced the first national lockdown in Italy (March 9 – May 4, 2020): 15.9% of the sample were in their first trimester, 36.4% in the second trimester, and 47.7% in the third trimester during the lockdown. These groups likely experienced differing levels of mobility restrictions, stressors, and access to outdoor environments. However, no significant differences were observed across these groups in levels of maternal trait anxiety, green space exposure, or infant *BDNF* DNAm (all *p*s > 0.30). Given these findings and the modest sample size, pandemic phase was not included as a covariate in the main analyses.

### Main analyses

**Fig. 3:**
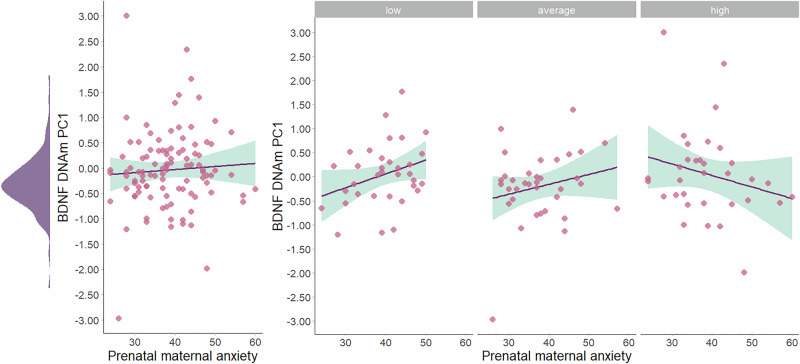
Association between maternal trait anxiety and infants’ *BDNF* DNAm PC1 as a function of antenatal exposure to green spaces within a 300 m buffer (*β* = −0.28 [95% *CI*. −0.49:−0.07], *p* = 0.008). For illustrative purposes, the association is plotted for infants antenatally exposed to lower ( − 1 SD), average, and higher ( + 1 SD) levels of green space within a 300 m buffer. These correspond approximately to 4.46–9.9%, 9.9–42.7%, and 42.7–86.8% of land classified as green space, respectively. Bands represent 95% CI.

Results of the hierarchical linear regression analyses predicting infants’ PC1 *BDNF* DNAm are presented in Table 3. A significant interaction emerged between maternal anxiety and total green space availability within a 300 m buffer on newborns’ PC1 *BDNF* DNAm (*β* = −0.28 [95% *CI*.−0.49:−0.07], *p* = 0.008), adjusting for infant sex and PM2.5. As illustrated in Fig. 3, simple slope analysis revealed that higher maternal anxiety was associated with increased *BDNF* DNAm in infants (*β* = 0.38, *p* = 0.014), but only at lower (i.e., −1 *SD*) green space exposure. In contrast, the association was not statistically significant (*β* = −0.18, *p* = 0.17) at higher levels ( + 1 *SD*) of green space availability. The model accounted for approximately 11.7% of variance in infant PC1 *BDNF* DNAm. The same analyses were replicated using 500 m and 1000 m buffers. For the 500 m buffer, the maternal anxiety × green space interaction yielded a *p*-value of 0.06, with the effect direction consistent with that detected at 300 m, although the overall model was not statistically significant (*p* = 0.14). Similarly, at the 1000 m buffer, the interaction was not statistically significant (*p* = 0.090). Analyses for newborns’ PC2 *BDNF* DNAm showed no significant independent or interactive effects of maternal anxiety and green spaces.

**Table 3:** Hierarchical linear regression analyses predicting infants’ *BDNF* DNA methylation PC1 and PC2 from maternal trait anxiety and green space exposure within a 300 m buffer.

	*BDNFm* PC1		*BDNFm* PC2	
	***β***	***p***	***β***	***p***
*Step 1*				
Sex	0.39	0.06	0.14	0.51
PM2.5	0.10	0.30	0.07	0.47
*Step 2*				
Maternal trait anxiety	0.06	0.53	−0.04	0.68
Green space	0.08	0.44	0.10	0.33
*Step 3*				
Trait anxiety X Green space	**−0.28**	**0.008**	0.08	0.44

Bold values indicate significant (p < 0.05) results.

For *BDNFm* PC1, *R*^*2*^ for Step 1 = 0.04, *F* = 2.08, *p* = 0.13 *ΔR*^*2*^ for Step 2 = 0.009, *p* = 0.63, *F* = 1.26, *p* = 0.29; *ΔR*^*2*^ for Step 3 = 0.07, *p* = 0.008, *F* = 2.54, *p* = 0.03.

For *BDNFm* PC2, *R*^*2*^ for Step 1 = 0.01, *F* = 0.40, *p* = 0.67; *ΔR*^*2*^ for Step 2 = 0.01, *p* = 0.56, *F* = 0.49, *p* = 0.74; *ΔR*^*2*^ for Step 3 = 0.01, *p* = 0.44, *F* = 0.51, *p* = 0.77.

### Supplementary analyses

A set of supplementary regression models explored specific associations between distinct green space indices (i.e., tree cover and low-growing vegetation) within the 300 m buffer and infant *BDNF* DNA methylation. The interaction between maternal anxiety and tree cover on newborns’ PC 1 *BDNF* DNAm was suggestive but did not reach statistical significance (*β* = −0.22 [95% *CI:*.−0.45, −0.01], *p* = 0.059) and the overall model was also not significant (*p* = 0.10). Likewise, the interaction between maternal anxiety and low-growing vegetation was significant (*β* = −0.22 [95% *CI:*−0.43, −0.01], *p* = 0.042), though the full model did not reach statistical significance (*p* = 0.11). Lastly, sensitivity analyses indicated that adjusting for PM2.5 did not affect the direction or significance of the associations. Furthermore, the interaction between maternal anxiety and green space remained statistically significant (β = −0.28 [95% CI: −0.50, −0.07], p = 0.009), after adjusting for maternal education and age, indicating that the observed effect is robust to adjustment for these sociodemographic characteristics.

## Discussion

This is the first study to examine the interactive effects of maternal anxiety during pregnancy and exposure to urban green spaces in predicting neonatal DNAm of the *BDNF* gene. Findings indicate that prenatal exposure to green space within a 300 m buffer mitigates the association between maternal trait anxiety and neonatal *BDNF* DNAm levels at 4 CpG sites, suggesting a potential neuroprotective role of natural environments during early development. Notably, the interaction was found soon after birth - likely independently of postnatal influences - and remained robust after adjusting for infant sex and prenatal PM2.5 exposure.

Pregnancy represents a critical window of susceptibility for the developing fetus to environmental challenges [2]. A wide range of prenatal individual-level factors, such as maternal symptoms of anxiety, depression and stress, and environmental-level influences, like air pollution, toxicants or residential green spaces, have been found to associate with bio-behavioral outcomes in offspring [6, 35, 36]. Furthermore, substantial evidence shows that these factors are independently linked to epigenetic changes in offspring [56]. However, the complex interplay among multiple simultaneous exposures across individual and environmental levels remains largely under-investigated. This study provides novel evidence of an interaction effect between prenatal maternal trait anxiety and green spaces in influencing neonatal *BDNF* DNAm. Specifically, higher *BDNF* DNAm at 4 CpG sites was observed in newborns exposed to both higher maternal trait anxiety and lower residential greenness. In contrast, the association was not significant with increasing availability of urban green spaces located very close to home. These findings raise important theoretical, methodological and clinical implications.

First, while maternal trait anxiety and urban green space alone were not associated with neonatal *BDNF* DNAm, a different picture appeared when their interaction was examined. This aligns with emerging literature on the effects of multiple antenatal exposures in shaping developmental trajectories [57]. Animal evidence [15, 58–60] suggests that *BDNF* DNAm is sensitive to the quality of the prenatal environment and may represent an epigenetic marker of antenatal exposures. The 11 CpG sites analyzed in this study are located within the promoter region upstream of exon IV of the BDNF gene, a regulatory region known to drive activity-dependent transcription. This promoter has been implicated in neuroplasticity, emotional learning, and stress regulation [61]. Recent animal studies have shown that methylation at this locus is associated with attenuated contextual fear expression [62], supporting its relevance as a stress-sensitive epigenetic target. Our findings suggest both prenatal individual- and environmental-level factors likely influence *BDNF* DNAm patterns, highlighting the need to move beyond unidimensional models of fetal programming, toward more ecologically valid multi-factorial approaches that integrate psychosocial and environmental exposures. The biological mechanisms underlying the buffering effect of green spaces in the link between maternal anxiety and *BDNF* DNAm are yet to be uncovered. Exposure to green spaces may offer a protective mechanism on newborns’ epigenome via several pathways. First, greenness may interact with physical and chemical environmental factors that are known to affect DNAm. For example, urban green space was negatively associated with PM2.5 levels in this sample. Urban vegetation can reduce harmful air particle concentrations through processes like adsorption and deposition [63, 64]. Prenatal PM2.5 exposure has been associated with altered neonatal DNAm patterns [65] and found to exacerbate the effects of maternal antenatal stress on infant DNAm of the *SLC6A4* gene [51]. However, it is noteworthy that all models were adjusted for PM2.5 and including it as a covariate did not change the direction and significance of the effects, suggesting that additional or complementary mechanisms might be at play. Second, exposure to natural environments promotes mental health, even during pregnancy [33, 34], and has been associated with reduced anxiety symptoms in the general population [66]. It is noteworthy that we did not detect any significant unadjusted correlation between green space availability and maternal trait anxiety levels. Trait anxiety is a relatively stable characteristic [25], selected here as an index of chronic stress during pregnancy [21, 44]. As such, it might be less sensitive to transient, state-level fluctuations that greenness could modulate. On the other hand, experimental studies showed that individuals with high trait anxiety might experience greater benefits, such as more reduced negative mood and improved positive mood, from brief green space exposure such as forest viewing [67] or walking in nature [68]. Although our study did not directly measure such transient effects, it remains plausible that living in greener neighborhoods contributes to overall stress mitigation and mood regulation in more vulnerable individuals, such as anxious pregnant women, thereby reducing stress-related epigenetic impacts on the fetus. Third, residential green space often inversely relates to neighborhood deprivation [69] known to affect newborns’ DNAm [70, 71]. Thus, green space measure may partly indirectly capture the effects of unmeasured contextual risk factors related to socioeconomic adversity. However, in our sample, no significant associations were observed between green space and key sociodemographic indicators, including parental age and educational attainment. While we cannot rule out the presence of more subtle or unmeasured neighborhood-level confounding, this also suggests that the observed interactive effect is unlikely to be entirely driven by socioeconomic factors. Lastly, exposure to green space has been linked to reduced inflammation, oxidative stress and stress-related hormones [31], all of which are known to influence epigenetic regulation. These pathways, though not directly measured in the current study, represent plausible mediating mechanisms for the observed interactive effects on fetal epigenetic programming.

From a methodological standpoint, this study emphasizes the need for antenatal stress research to account for broader environmental aspects and multiple exposures. Overlooking these factors might lead to misleading findings and explain inconsistencies in the literature. Furthermore, we explored the effects of green spaces over different buffer sizes (i.e. 300, 500 and 1000 m) and reported a statistically significant interaction of maternal anxiety and green spaces on newborn’s *BDNF* DNAm only when a 300 m buffer was employed. When using larger buffers (500 and 1000 m), the interaction effects approached significance but did not reach statistical thresholds (*p* = 0.060 and *p* = 0.090, respectively), and the full models were not significant. Notably, the direction of the effect remained consistent across all distances, suggesting a proximity-specific effect that is theoretically and empirically plausible in light of previous environmental exposure research. Although replication is needed, this result aligns with prior research indicating that proximal greenness is most strongly associated with health outcomes [45] and emerging evidence showing stronger associations between green space assessed within the 100 m buffer and DMRs in cord blood [41]. It is also important to mention that this sample was enrolled during the COVID-19 pandemic in Italy, when stringent public health restrictions limited access to outdoor spaces and public parks. While green spaces within 1000 m are typically thought to promote health and well-being by encouraging access to parks, physical outdoor activity or social contacts, such pathways might have been limited during the pandemic. In contrast, green space within closer proximity might have not only reduced the impact of environmental stressors such as air pollution, heat and noise, but also reduced maternal stress through direct views of nature, as supported by previous studies [32, 72]. Supplementary analyses investigated the role of specific types of vegetation and biodiversity (i.e., tree cover and low-growing vegetation) within the 300 m buffer. Although some interactions between maternal anxiety and these distinct vegetation types showed trends in the expected direction, the full models did not reach the statistical significance and should be interpreted with caution. Future studies with larger samples are needed to disentangle the relative contributions of urban green considering different elements, such as the green structure, naturalness, size or accessibility, and to identify the features of green space that might be more protective during pregnancy.

The clinical implications of these findings are substantial. Heightened DNAm of the *BDNF* gene has been reported across various neurodevelopmental and psychiatric disorders [14, 15, 17, 18, 73], and early evidence suggests that elevated *BDNF* DNAm at birth may increase susceptibility to later psychopathology [21]. Our results highlight a potential avenue for early intervention. Identifying pregnant women with elevated trait anxiety and limited access/exposure to green spaces could allow for targeted interventions promoting nature contact. This could involve integrating “green prescriptions” into prenatal care, such as encouraging time spent in parks, or natural settings, or promoting community-based initiatives that improve the accessibility and quality of urban green spaces for expectant mothers. Encouraging nature engagement during pregnancy may represent a low-cost, scalable, and non-pharmacological strategy to support maternal wellbeing and foster fetal development. In this vein, our findings underscore the potential for urban planning and environmental design as key components of public health strategies to promote healthy child development from conception onwards. Enhancing green infrastructure, particularly in socioeconomically disadvantaged areas, may contribute to reducing intergenerational health inequalities linked to prenatal stress. While green space exposure is unlikely to serve as a stand-alone solution to the multifactorial etiology of mental health disorders, it may constitute a meaningful component of a broader ecological prevention strategy. Future trials should assess whether increasing pregnant individuals’ access to and engagement with natural environments leads to measurable improvements in maternal stress physiology and epigenetic outcomes in offspring.

Several limitations of this study warrant consideration. First, results are based on a relatively small community sample enrolled during the pandemic, thus limiting generalizability of findings to high-risk populations or different time periods. Second, we assessed neonatal *BDNF* DNAm using buccal cells. While some studies suggest that salivary samples can serve as a reasonable proxy for brain tissue methylation [74], the exact relationship between epigenetic variation in peripheral tissues and brain-specific epigenetic changes remains unclear, thus the functional relevance of these findings cannot be definitively established. Third, we focused on a specific region of the *BDNF* gene; it is important to recognize that several other genomic regions might be impacted by exposure to antenatal adversity. Furthermore, gene polymorphisms are likely to moderate this impact and warrant inclusion in future studies. Fourth, maternal antenatal stress was measured using a single validated self-report measure of trait anxiety. This approach may not capture the full spectrum of antenatal stressors that could influence fetal epigenetics. Future research would benefit from incorporating multiple measures of maternal stress. Fifth, the effect size observed for the interaction between maternal anxiety and green space was modest, and the robustness of the effect varied across different spatial buffer distances. While the direction of the interaction remained consistent, statistical significance was primarily observed for green space in close proximity to the residence (300 m), suggesting a proximity-specific effect that should be interpreted with caution and explored further in larger samples. Sixth, while we accounted for key sociodemographic and environmental factors, we acknowledge that the COVID-19 pandemic context may have differentially shaped participants’ experiences during pregnancy. Our exploratory analyses suggest that nearly half of the sample experienced the lockdown during their third trimester, while others were in earlier stages. Although we did not find significant differences in anxiety, greenness, or DNAm levels between these groups, future research should further investigate how pandemic-related disruptions and public health measures may have influenced prenatal exposures and developmental outcomes. Lastly, results are correlational, and causality cannot be inferred.

## Conclusions

While mechanisms underlying the observed effects deserve further investigation, this study provides novel evidence for the buffering effect of prenatal green space exposure on the association between maternal trait anxiety and neonatal *BDNF* DNAm. Our results suggest that exposure to natural environments during pregnancy may play a neuroprotective role, potentially mitigating the epigenetic impact of maternal stress on the developing fetus. These findings advance understanding of how environmental-level and individual-levels factors interact to influence early life programming. Future research should explore the long-term functional consequences of the observed epigenetic modifications and their potential links to later cognitive, emotional, and behavioral outcomes in children. Importantly, these findings underscore the potential for green space exposure to serve as a public health intervention to reduce the intergenerational transmission of risk for mental health issues. Enhancing urban green infrastructure and promoting contact with natural environments from pregnancy onward may offer a scalable strategy to foster resilience from the very beginning of life.

## Supplementary information

Supplemental Material

## Data Availability

The raw data supporting the findings of this study will be made available on Zenodo.
