# Supplementary material for: How nature nurtures: prenatal exposure to green space buffers the effects of maternal stress on neonatal BDNF methylation
Source: Mol Psychiatry. 2025 Dec 13;31(5):2418–26. doi: 10.1038/s41380-025-03379-1 (PMC13099633; doi:10.1038/s41380-025-03379-1)
Supplement: Supplementary file 1 — Supplemental Material [file 41380_2025_3379_MOESM1_ESM.docx]

**SUPPLEMENTARY MATERIALS**

SUPPLEMENTARY METHODS

*Infant BDNF DNAm analysis*

We analyzed one target region of the *BDNF* gene containing 11 CpG sites (positions detailed in Supplementary Figure 1). Methylation assessment was performed at the Molecular Biology Lab of the Scientific Institute IRCCS E. Medea. Methylation levels were determined in DNA using bisulfite modification followed by PCR amplification and NGS. Genomic DNA was extracted from 0.2 ml of each sample using the GenElute Blood Genomic DNA kit (Sigma). Bisulfite conversion was performed on 500 ng of genomic DNA using the EZ DNA methylation kit (ZymoResearch, Inc., Irvine, CA, USA). Primers were designed using Bisulfite Primer Seeker. A specific tail was added to each primer in order to allow synthesis and sequencing of SureSelect-like libraries of methylated fragments. Primary PCR-amplification was performed on 20 ng of bisulfite-treated DNA using Taq Gold (Life Technologies, Inc.). Cycling comprised 5 min preactivation at 95°C, followed by 35 cycles of 94°C denaturation for 15 s, 58°C annealing for 20 s, 72°C elongation for 1.5 min. All PCR products were checked on 2% agarose gels and treated with Ilustra Exo Pro-STAR (GE Healthcare) to eliminate unincorporated primers. Secondary PCR was conducted on each sample using a SureSelect Custom Amplicon Index Kit (Agilent) containing eight forward (i5) and 12 reverse (i7) index primers. Optimal annealing temperature (68°C) and number of PCR cycles were experimentally determined. Cycling comprised 5 min pre-activation at 95°C, followed by 16 cycles of 94°C denaturation for 15 s, 68°C annealing for 20 s, 72°C elongation for 1 min. All PCR products were checked on 2% agarose gel, and approximately equimolar amounts of each product were purified using AMPure XP beads (Beckman Coulter) and pooled (up to 96 libraries/pool). The purified pooled libraries were quantified on a Bioanalyzer 2100 (Agilent) and sequenced on a NextSeq 500 (Illumina) using a v2 Reagent kit, 2x150 cycles. Paired ends reads from each sample were independently aligned to all the reference sequences by a parallel striped Smith–Waterman algorithm. Only paired reads that aligned coherently to the reference sequence were retained. Adaptors were trimmed with TrimGalore-0.6.6; alignment was performed using Bismark_v0.20.0; CpG methylation counting was conducted with IGVTools. At each CpG site in each sequence, the four base frequencies were evaluated and reported along with the C-to-T percentage. Average read depth was 28370 reads. Samples with less than 1000 reads were excluded.

*Green Space Exposure Assessment*

Residential addresses provided by participants were geocoded to latitude and longitude coordinates using the Geocode Tool in ArcGIS Pro, referencing the WGS84 coordinate system. Green space availability in proximity to each residence was assessed using the CLCplus Backbone 2021 dataset, a harmonized, pan-European land cover inventory with detailed classification for the reference year 2021. To quantify surrounding greenness, circular buffers with radii of 300 m, 500 m, and 1000 m were generated around each geocoded residence. These distances were selected based on prior evidence showing that residential greenness measured within 500–999 m buffers is significantly associated with physical and mental health outcomes (Gascon et al., 2018). Within each buffer, the percentage of land cover was extracted using GRASS GIS 8. Green space was defined based on the following CLCplus categories:

- 2: Woody – Needle-leaved trees
- 3: Woody – Broadleaved deciduous trees
- 4: Woody – Broadleaved evergreen trees
- 5: Low-growing woody plants (e.g., bushes, shrubs)
- 6: Permanent herbaceous vegetation
- 8: Lichens and mosses

Land classified as sealed surfaces (Category 1) was included to calculate artificial land cover.
Based on these categories, three indices were computed for each buffer:

- Tree cover: Categories 2, 3, 4
- Low-growing vegetation: Categories 5, 6, 8
- Total green space: Sum of all six green categories
- Artificial surface: Category 1

This classification is consistent with recent methods distinguishing high (>3 m) and low (<3 m) vegetation (Garber et al., 2024; Vos et al., 2024).
All participants reported a single, unchanged residential address during pregnancy, and thus no address variation was included in analyses.

SUPPLEMENTARY TABLES

**Supplementary Table 1.** Principal component analysis (PCA) on infants’ *BDNFm* conducted among the 11 CpG sites. Loadings on the respective principal components (PC) are reported in bold. Loadings < .300 are not reported.

| *BDNFm* sites | **Principal Components** | | | |
| --- | --- | --- | --- | --- |
|  | **PC1** | **PC2** | **PC3** | **PC4** |
| CpG 01 | **.599** | .481 |  |  |
| CpG 02 | **.778** |  |  |  |
| CpG 03 | **.860** |  |  |  |
| CpG 04 |  | .430 | **.758** |  |
| CpG 05 |  | **.758** | .392 |  |
| CpG 06 |  |  | **.754** |  |
| CpG 07 |  |  |  | **.899** |
| CpG 08 | **.813** |  |  |  |
| CpG 09 |  |  | .542 | **.650** |
| CpG 10 | .321 | **.769** |  |  |
| CpG 11 |  | **.750** |  | .416 |

Note. “Varimax” rotation was used

**Supplementary Table 2.** Bivariate correlations among study variables

|  |  | 1 | 2 | 3 | 4 | 5 | 6 | 7 | 8 | 9 | 10 | 11 |
| --- | --- | --- | --- | --- | --- | --- | --- | --- | --- | --- | --- | --- |
| 1 | Maternal trait anxiety |  |  |  |  |  |  |  |  |  |  |  |
| 2 | Maternal age | 0.18 |  |  |  |  |  |  |  |  |  |  |
| 3 | Maternal education | 0.16 | 0.18 |  |  |  |  |  |  |  |  |  |
| 4 | Gestational age | 0.06 | -0.16 | 0.03 |  |  |  |  |  |  |  |  |
| 5 | Birth weight | 0.15 | 0.03 | 0.08 | **0.43***** |  |  |  |  |  |  |  |
| 6 | Apgar score 1min | -0.02 | -0.14 | 0.15 | 0.16 | 0.07 |  |  |  |  |  |  |
| *7* | Total PM 2.5 | 0.08 | -0.00 | -0.02 | 0.01 | -0.03 | -0.12 |  |  |  |  |  |
| *8* | Total green space 300m | -0.08 | 0.02 | -0.01 | -0.13 | -0.07 | 0.09 | **-0.27**** |  |  |  |  |
| 9 | Total green space 500m | -0.10 | 0.05 | -0.05 | -0.08 | -0.02 | 0.07 | **-0.34***** | **0.91***** |  |  |  |
| 10 | Total green space 1000m | -0.03 | 0.06 | -0.00 | -0.03 | 0.06 | 0.12 | **-0.45***** | **0.68***** | **0.88***** |  |  |
| 11 | *BDNF DNAm* PC1 | 0.06 | 0.14 | 0.03 | 0.02 | 0.03 | 0.07 | 0.06 | 0.11 | 0.07 | 0.02 |  |
| 12 | *BDNF DNAm* PC2 | -0.10 | 0.06 | 0.08 | 0.01 | 0.01 | 0.16 | 0.09 | 0.03 | 0.01 | -0.05 | 0.09 |

* p<.05; **p<.01; ***p<.001; Note. Bold values indicate significant (p<.05) results.

SUPPLEMENTARY FIGURES


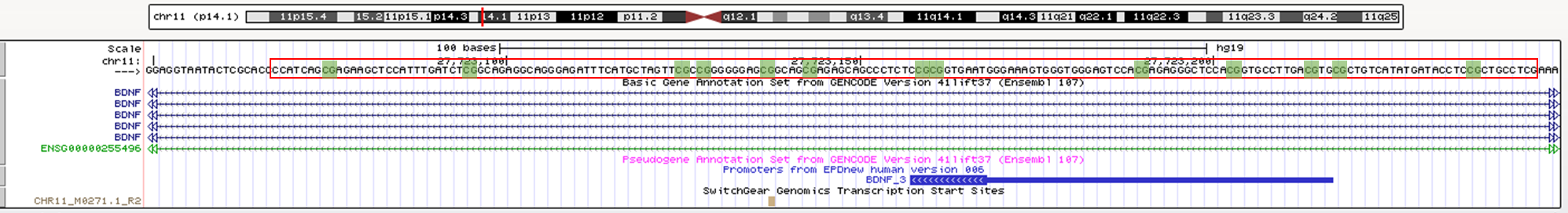


**Supplementary Figure 1.** Schematic representation of the BDNF gene with the target sequence investigated (chr11: 27,723,096–27,723,219; UCSC human genome browser GRCh37/hg19)
